# A Thank You Note to Our Reviewers

**DOI:** 10.1002/jgf2.70079

**Published:** 2025-11-11

We would like to express our deepest gratitude to all the individuals who have provided their valuable time and expertise to support the *Journal of General and Family Medicine*. The Editorial Board wishes to acknowledge with gratitude the following Reviewers for reviewing manuscripts during the past year.

Abe, Kazuhiro

Achterberg, Wilco

Aihara, Hidetoshi

Akimoto, Masatoshi

Akiyama, Yutaro

Almuammar, Sarah

Ando, Takayuki

Ang, Gary

Ang, Yee

Aoki, Takuya

Arai, Hidenori

Araki, Kazuo

Asakawa, Shoko

Asakura, Kentaro

Ayano, Masahiro

Ayusawa, Mamoru

Azuma, Teruhisa

Barary, Mohammad

Baz, Sarah

Borrow, Ray

Chang, Yu‐Ting

Chen, Yang W.

Chiaranai, Chantira

Cho, Mi‐Kyoung

Chojin, Yasuo

Cumming, Jacqueline

Daley, Stephanie

Devaraj, Navin Kumar

Dohi, Eisuke

Endo, Takeshi

Fujihara, Manabu

Fujii, Hirofumi

Fujikawa, Hirohisa

Fujino, Naoya

Fujita, Koji

Fujiwara, Motoshi

Fujiwara, Shinji

Fukuchi, Takahiko

Fukui, Sho

Goda, Ken

Gomi, Harumi

Goto, Tadao

Hagiwara, Shotaro

Hamada, Hisayuki

Hamada, Shota

Hamada, Shuhei

Hamamura, Toshitaka

Haneke, Eckart

Harada, Taku

Harada, Yukinori

Haraguchi, Masahiro

Haraguchi, Mizuki

Haruta, Junji

Hase, Ryota

HashimotoKunihiko

Hashizume, Naoki

Hasunuma, Naoko

Hattori, Tadashi

Higashimoto, Yuji

Hinata, Yuki

Hirai, Jun

Hirayama, Yoko

Hirose, Hideo

Hirose, Masahiro

Hokama, Akira

Horibata, Ken

Horinouchi, Noboru

Hsiao, Po‐Jen

Hsu, Chao‐Kai

Ichikawa, Shuhei

Igarashi, Shun

Iguchi, Seitaro

Ikeda, Kotaro

Ikeda, Takaaki

Ikuno, Masashi

Imanaga, Teruhiko

Inoue, Kenji

Inoue, Machiko

Inoue, Yoshie

Ishi, Mitsuaki

Ishibashi, Hiroki

Ishikane, Masahiro

Ishikawa, Yukiko

Ishimaru, Naoto

Ishimaru, Hiroyasu

Ishizuka, Kosuke

Isik, Arda

Isse, Naohi

Iwamuro, Masaya

Iwata, Hiroyoshi

Kako, Mayumi

Kamimoto, Minako

Kanakubo, Yusuke

Kaneko, Makoto

Kanke, Satoshi

Kanno, Tetsuya

Kano, Yasuhiro

Kanzawa, Yohei

Kashiura, Masahiro

Kataoka, Hitomi

Kataoka, Yuki

Katayama, Kohta

Kawamoto, Ryuichi

Kawashima, Atsushi

Kawauchi, Hideyuki

Kenzaka, Tsuneaki

Kikukawa, Makoto

Kimura, Takuma

Kishi, Tomomi

Koda, Masahide

Koike, Soichi

Kojima, Taro

Kokkinakis, Ioannis

Komagamine, Junpei

Kondo, Satoshi

Kosaka, Takeo

Kubota, Kazumi

Kudo, Takahiro

Kume, Yu

Kuroda, Kaku

Kuroda, Moe

Kuwahara, Susumu

Little, Sahoko

Longenecker, Randall

Maeno, Tetsuhiro

Masumoto, Shoichi

Matsumoto, Monami

Matsumoto, Tomohiro

Matsumura, Shin

Matsumura, Shinji

Matsunaga, Nobuaki

Mezawa, Hidetoshi

Midlöv, Patrik

Miller, Eden

Minato, Mami

Mitsuzawa, Sadaki

Miyagami, Taiju

Miyamichi, Ryosuke

Miyamoto, Yuki

Miyata, Yasushi

Miyazaki, Jun

Miyazawa, Asako

Mizukami, Takayuki

Mizumoto, Junki

Mizutani, Naoya

Momosaki, Ryo

Mori, Tomoari

Morikawa, Toru

Morishita, Motoyoshi

Motomura, Kazuhisa

Nagao, Noriko

Nagasaki, Kazuya

Nagoshi, Kiwamu

Nakajima, Nobuyuki

Nakayama, Gen

Narita, Masashi

Narumoto, Keiichiro

Niedermaier, Tobias

Nishida, Sakiya

Nishiguchi, Sho

Nishioka, Daisuke

Nishioka, Hiroaki

Noguchi, Taiji

Noguchi‐Watanabe, Maiko

Nojima, Tsuyoshi

Noshioka, Daisuke

Ohira, Yoshiyuki

Ohta, Ryuichi

Okada, Tadao

Okayama, Masanobu

Okazaki, Kentaro

Okuyama, Kenta

Omura, Daisuke

Onda, Mitsuko

Onizawa, Hideo

Ono, Ryohei

Ota, Kazuhiro

Otsuka, Fumio

Otsuka, Yuki

Oura, Makoto

Ozaki, Makiko

Ozone, Sachiko

Pileggi, Claudia

Priego‐Parra, Bryan Adrián

Romagnoli, Katrina M.

Sada, Ken‐ei

Sakamoto, Hirotsugu

Sanada, Yuichi

Sato, Mikiya

Satoh, Kasumi

Satoi, Yoshinao

Sawada, Akinari

Sechi, Gian Pietro

Shibata, Ayako

Shikino, Kiyoshi

Shimada, Kazuyuki

Shimizu, Akio

Shimizu, Hiroshi

Shimizu, Yoichi

Shinohara, Koh

Son, Daisuke

Sugawara‐Mikami, Mariko

Sugaya, Nagisa

Sugiyama, Takehiro

Sun, Yu

Suzuki, Masao

Takahashi, Noriyuki

Takamura, Akiteru

Takayama, Shin

Takayashiki, Ayumi

Taketani, Takeshi

Takeuchi, Jiro

Tamura, Haruka

Tanaka, Kosuke

Taniguchi, Tomohiro

Tanizaki, Ryutaro

Terabe, Hiroya

Tetsuka, Syuichi

Tillmann, Hans L.

Tokinobu, Akiko

Tokumasu, Kazuki

Tsuchida, Tomoya

Tsuji, Yoshihisa

Tsujinaka, Shingo

Uchida, Takuro

Ueno, Tsukasa

Ukawa, Shigekazu

Usami, Jun

Van Leuven, Sander I

Wada, Mikio

Watanuki, Satoshi

Yabuki, Taku

Yahata, Shinsuke

Yamada, Hiroyuki

Yamaguchi, Yoshiko

Yamamoto, Shuhei

Yamamoto, Shungo

Yamamoto, Yu

Yamanashi, Hirotomo

Yanagawa, Youichi

Yodoshi, Toshifumi

Yokoh, Hidetaka

Yokokawa, Hirohide

Yokota, Yuya

Yokoya, Shoji

Yoshida, Eriko

Yoshimi, Kanako

Yoshimoto, Kiyomi

Yoshimura, Yoshihiro

Zukeran, Sota

Zullo, Angelo

